# Exploring Unobserved Heterogeneity in Cyclists’ Occupying Motorized Vehicle Lane Behaviors at Different Bike Facility Configurations

**DOI:** 10.3390/ijerph19020792

**Published:** 2022-01-11

**Authors:** Lei Zhang, Shengrui Zhang, Bei Zhou, Yan Huang, Dan Zhao, Shuaiyang Jiao

**Affiliations:** 1 College of Transportation Engineering, Chang’an University, Xi’an 710064, China; zhanglei2018@chd.edu.cn (L.Z.); bzhou3@chd.edu.cn (B.Z.); yanhuang@chd.edu.cn (Y.H.); dzhao@chd.edu.cn (D.Z.); jiaoshuaiyang@chd.edu.cn (S.J.); 2 School of Civil and Transportation Engineering, Henan University of Urban Construction, Pingdingshan 467036, China; 3 Procurement and Bidding Office, Northwest University, Xi’an 710069, China

**Keywords:** cyclists occupying motorized vehicle lane behaviors, field data, unobserved heterogeneity, random parameter logit model

## Abstract

Cyclists occupying motorized vehicle lanes disrupt road traffic order and increase collisions. Exploring the contributing factors could help develop countermeasures to regulate such behaviors. The purpose of this study is to explore the intrinsic features influencing the behavior of cyclists in occupying motorized vehicle lanes at different bicycle facilities. We investigated a total of 34,631 cycling behavior samples in the urban area of Pingdingshan, China. A Bayesian random parameter logit model was used to account for the unobserved heterogeneous effects. The experimental results of all bike facilities demonstrate that the bike type, dividing strip type, bike lane width, temporary on-street parking, and whether it is a working day significantly affect cyclists’ occupying motorized vehicle lane behaviors. Factors associated with unobserved heterogeneity are age, barriers dividing strip, vehicle lane numbers, bike volume, vehicle volume, and daily recording time intervals. Comparing the estimated model of five type bike lane facilities across different dividing strips, we find that cyclists have a significantly different occupying probability and the heterogeneity factors of the various bike facilities also have their focus. When the non-motorized road conditions become more open, the cyclist behavior becomes more random and the heterogeneity factors become broader.

## 1. Introduction

With the development of the economy of China and society, motor-driven oriented traffic has led to tremendous traffic congestion pressure on urban traffic. However, riding bicycles as a fast, low-cost, and flexible mode of transportation meets the strong demand of the working class for travel, which has an increasing advantage in the growth of the user market [1]. At the same time, encouragement from government transportation authorities has also stimulated riding [2]. In recent years, the number of non-motor vehicles has increased. According to the China Bicycle Industry Conference 2019 and the Ministry of Public Security Statistics, as of September 2020, the number of motor vehicles in China has reached 365 million, and the number of bicycles has reached nearly 400 million, out of which nearly 300 million are electric two-wheelers [3]. Non-motorized vehicles, particularly electric two-wheelers, have become an essential mode of transportation for residents traveling short and medium distances. The rapid growth in the number of electric two-wheelers presents new challenges to transportation systems [4]. New modes of transport changes the manner of mobility [5], which may lead to remarkable changes in cyclist behavior compared to the past [6].

In addition, there is a severe mismatch between the rapid growth of non-motorized vehicles and the construction of motor vehicle-oriented transportation facilities [7]. Competition between motorized and non-motorized vehicles for limited road space has intensified, triggering widespread conflict, especially at road sections with large traffic volume [8,9,10]. Illegal behaviors such as that of cyclists occupying the motorized vehicle lane are very common, raising a series of safety issues.

According to the National Bureau of Statistics of China, non-motorized vehicles accounted for 11.7% of traffic accidents in 2019 [11]. The most common type of accident involving non-motorized vehicles is a collision between a non-motorized vehicle and a motor vehicle [12]. Most of the accidents involve cyclists occupying motorized vehicle lane behavior. Moreover, the contradictions caused by occupying motorized vehicle lanes for cycling are also increasingly alarming. Why are the riders riding on the motorized vehicle lanes? What factors are associated with these behaviors? There is a dearth of research focusing on the factors that influence cyclists occupying motorized vehicle lane.

The primary objective of this study is to explore the intrinsic features that influence cyclists occupying motorized vehicle lane behavior at different bicycle facilities. The random parameter logit model was constructed to account for the unobserved heterogeneous effect. The results of the study can provide useful countermeasures for regulating cyclists’ behavior and bike lane design; moreover, we believe the findings can offer suggestions to traffic authorities regarding alleviating traffic conflicts and improving traffic safety.

The remainder of this paper is organized as follows. Section 2 presents the literature review. Section 3 describes the data collection on cyclists’ occupying motorized vehicle lanes behavior. Section 4 explains the methodology of the random parameter logit model and so the fifth section explains the results and discussion from the estimated model. Finally, the “Conclusions” section highlights the major findings and limitations of the research.

## 2. Literature Review

With the rapid growth in the number of non-motorized vehicles and changes in bike type, the riding behavior of cyclists has become increasingly complex. Studies have revealed that riding on vehicle lanes, red-light running, and failing to obey traffic signs are the main factors leading to accidents [13]. Non-motor vehicle violations account for 60% of fatal non-motor vehicle accidents [14]. A series of studies began to explore risky riding behaviors. Schleinitza et al. [15] used generalized estimating equations from the characteristics of non-motorized vehicle models and infrastructure to explore the behavior of cyclists running red lights. Gkritza et al. [16] studied the effects of road conditions, geographic location, and weather on helmet use by cyclists. Ma et al. [13] presented 12 risky riding behaviors and noted that illegal lane-taking and riding with others resulted in the highest likelihood of traffic accidents. Parkin et al. [17] established the model between the perceived cycling risk and route acceptability and assessed the potential demand for cycling. Existing research on risky riding behaviors mainly focuses on red-light running, not wearing helmets, risk perception, and so on. There are few empirical investigations on cyclists occupying motorized vehicle lanes behavior which brings a higher proportion of accidents.

Previous studies have shown that bike facility configuration plays a significant role in the behavioral choices of cyclists [9,18,19,20]. Some studies indicated that people feel safest when riding in physically-divided strip bike lanes; the appropriate infrastructure encouraged people to ride freely. On the contrary, people feel unsafe when riding in mixed traffic, where riding is restricted [21]. Strauss et al. [9] proposed a methodology to estimate bicycle volumes and cyclist injury risk throughout the entire network of road segments and intersections, justifying the benefits of cycle tracks. Nikiforiadis et al. [22,23] explored the pedestrians–cyclists shared use space and quantified the impact of interactions; moreover, the authors highlighted concerns regarding conflicts and delayed passing in access. Kaplan et al. [10] proposed the joint model of frequency and severity of cyclist–motorist collisions, and emphasized the focus on bicycle paths to improve road design and traffic management. Torrisi et al. [24] believed that infrastructure construction can promote the use of sharing bikes. Moreover, the setting of management facilities also had a significant impact on cycling. Pedestrians and on-street parking facilities significantly increase cycling conflicts [8]. These studies have attempted to link traffic facilities to cycling behavior, which has provided us with inspiration [25]. To establish a more robust understanding of the behaviors of cyclists and determine the influence of infrastructure design and operations on them, we try to explore the factors influencing the behavior of cyclists in occupying motorized vehicle lanes at different bicycle facilities.

Several modeling procedures have been applied in the past to explore the behavior of cyclists. Binary logit models were used to explore non-motorized rider-risk behaviors [26]. Ordered logit models were used to analyze the relationship between riding behavior and accident severity [27]. Generalized estimating equations were used to explore the relationship between cyclist behavior and different traffic environments [15]. However, these traditional models do not allow for different outcomes for the explanatory variables. In reality, each individual outcome responds differently to explanatory variables and thus cannot be considered fixed [28]. Unobserved heterogeneity across individuals influences the likelihood of the behavior of cyclists [29]. There are some unobserved factors affecting the behavior of individual cyclists, for which comprehensive data collection is sometimes difficult. For instance, conventional models may include gender as a predictor of red-light running behavior. However, even within the same gender group, the likelihood of a cyclist running a red light varies according to height, weight, or other physical and psychological factors [30]. If the unobserved heterogeneity is ignored, the model will be inaccurate, resulting in biased parameter estimates and incorrect inferences [31]. Therefore, some recent studies have highlighted the possibility of using a random parameter logit model [32,33]. Thus, analyzing the behavior of cyclists to address unobserved heterogeneity is essential.

To the best of our knowledge, there is a dearth of research on cyclists occupying motorized vehicle lanes behavior. Only Ma et al. [34] used a questionnaire to reveal the potential relationship between the personal characteristics of electric two-wheeler riders and illegal occupancy of motorized vehicle lanes, which demonstrated that individuals have different probabilities of behavior selection. Zhang et al. [14] investigated the behavioral characteristics of cyclists occupying motorized vehicle lanes; however, the study did not explore cyclist-occupying behavior at different bicycle facilities, and the heterogeneity of individuals cyclists’ behavior was ignored. Therefore, based on the research experience of relevant authors mentioned earlier, we endeavored to explore such large-scale violations from a data-driven perspective and examine the mechanism of this behavior in all relevant bicycle facilities types in China. Moreover, a flexible and robust framework is necessary to capture the heterogeneity in the behavior of cyclists. We aim to address these challenges, and the goals of this research are as follows:

1. To estimate a random parameter logit model for the behavior of cyclists in occupying motorized vehicle lanes;
2. To determine the effects of individual characteristics, geometric road design, environmental characteristics, and traffic variables on the behavior decisions of cyclists using risk factor analysis and simulated probability; and
3. To demonstrate the effect of the different bike facility configurations on the behavior of cyclists occupying motorized vehicle lanes that will assist traffic management authorities in developing appropriate countermeasures.

## 3. Data

### 3.1. Definition of Cyclists Occupying Motorized Vehicle Lanes

**Figure 1:**
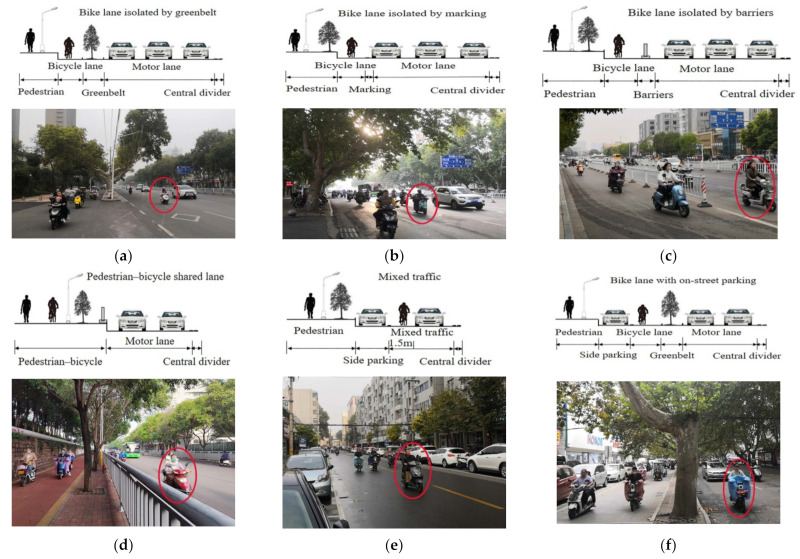
Different types of bike facility configurations: (**a**) Bike lane isolated by greenbelt; (**b**) Bike lane isolated by marking; (**c**) Bike lane isolated by barriers; (**d**) Pedestrian–bicycle shared lane; (**e**) Mixed traffic; (**f**) Bike lane with on-street parking. Red marks indicate that cyclists occupy motorized vehicle lanes.

In this survey, cyclists occupying the motorized vehicle lane are defined as cyclists crossing or touching the separation boundary between non-motor and motor vehicles, regardless of the duration. This can be seen in Figure 1a–d. In the mixed traffic, a cyclist who rides on the right side of the road, but goes further than 1.5 m from the face of the curb is considered to occupy the motorized vehicle lane. This is defined by the interpretation of the Road Traffic Safety Law of the People’s Republic of China. Non-motorized vehicles are to be driven within 1.5 m to the left of the right edge line of the carriageway on roads without non-motorized vehicle lanes. This can be seen in Figure 1e.

### 3.2. Data Collection

#### 3.2.1. Data Investigation

A field survey of cyclists’ occupying motorized vehicle lanes behaviors (COMB) was implemented in Pingdingshan, Henan Province, where riding is an essential way for residents to get around. Videotaping the behavior of bicyclists who were unaware that they were being observed would be ideal, and the survey was conducted on five weekdays and one non-working day in September and October 2020. The weather was mainly sunny and cloudy, with occasional light rain. The daily recording time intervals included the morning peak (7:30–8:30), afternoon peak (14:00–15:00), and evening peak (17:30–18:30) periods.

According to the dividing strip types between the motor vehicle and non-motor vehicle lanes, five types of typical urban bicycle facilities were selected for data collection to ensure a variety of configurations and characteristics. The characteristics of the selected sites are listed in Table 1.

The principles for the selection of the five types of bike facilities are as follows: (1) Non-motor and motor lanes were isolated by a greenbelt (with on-street parking or without on-street parking). (2) Non-motor and motor vehicle lanes were isolated by marking (with on-street parking or no on-street parking). (3) Non-motor and motor vehicle lanes were isolated by barriers. (4) Pedestrian–bicycle shared lane. (5) Mixed traffic, non-motorized lanes, and motor vehicle lanes were mixed. The types of bicycle facilities used are shown in Figure 1.

#### 3.2.2. Data Extraction and Description

Two video cameras were set up in the field for data collection. One was placed next to the roadway to film the entire cycling process of the riders at the cross-section. Meanwhile, another camera was used to observe the traffic volume on the segment. Videos were reviewed in the laboratory for data reduction. Four laboratory professionals identified and encoded information about each cyclist in the video. Whether the cyclist occupies the motorized vehicle lane shall be determined following the standard defined in Section 3.1. Finally, 34,631 cycling samples and four types of necessary data were extracted and recorded; Table 2 summarizes the descriptive statistics of the data.

**Figure 2:**
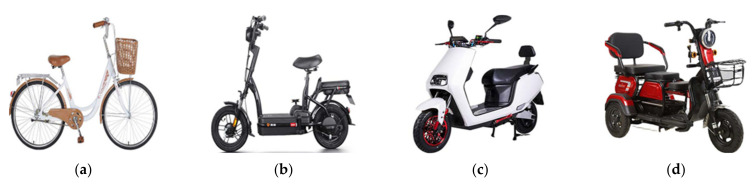
Bike types: (**a**) Bicycle; (**b**) E-bike; (**c**) E-scooter; (**d**) Electric tricycle.

Individual characteristics. For each rider who arrived at the cross-section, the research team recorded the crossing legality (that is, occupying the motorized vehicle lane or not), gender, age by visual inspection, and bike type. The riders were divided into three groups: young, middle-aged, and old. The bike type included a conventional bicycle, electric bicycle, scooter-style bicycle, and light electric tricycle. These are shown in Figure 2. Among them, several, such light electric tricycles, run on bike lanes; thus, we have also conducted surveys and statistics.

Road geometric design. The geometric design of the road includes the bike lane width, vehicle lane number and dividing strip types between the non-motorized lane and the motorized vehicle lane.

Traffic conditions. The traffic conditions include bike volume and vehicle volume. On-street and temporary on-street parking in bike lanes were also considered. Traffic volume was recorded every 5 min. Bike volume was converted to veh/(5 min·m). Vehicle volume was converted to veh/(5 min·lane).

Environment and other conditions. Weather, daily recording time intervals, and manned riding.

On the cross-section, a total of 34,631 cycling behaviors were observed and used to construct the models, out of which 9917 were cyclists occupying motorized vehicle lanes, and 24,714 were cyclists with normal cycling behaviors. The proportion of cyclists occupying motorized vehicle lanes was 28.64%.

## 4. Methodology

### 4.1. Random Parameter Logit Model

Cyclists occupy the motorized vehicle lane in a random manner. The conventional method for this alternative, as a two-class outcome, is generally analyzed using a binomial logit model; however, the logit model does not consider unobserved heterogeneity of the different observations. It is possible that the parameter estimates of the model will result in biased estimates.

**Figure 3:**
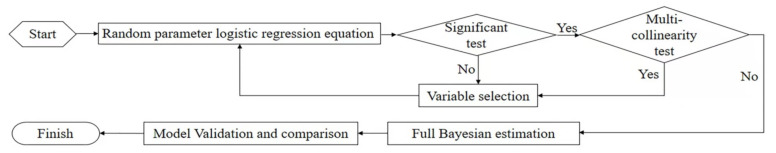
Diagram of Bayesian random parameter logit model.

This study proposes a Bayesian random parameter logit model to investigate the risk factors affecting cyclists occupying motorized vehicle lanes to account for the unobserved heterogeneity. In comparison with the fixed-parameter standard logit model, the random parameter logit model allows all parameters to vary randomly across observations. It considers the unobserved heterogeneity of explanatory variables in cyclists occupying motorized vehicle lanes. Thus, more features in the data can be extracted, and the accuracy of the model can be improved. A diagram for this methodology is shown in Figure 3.

The random parameter logit model was developed as expressed in Equations (1) and (2) as follows:

(1) $$y_{i}∼Bernoulli\left(p_{i}\right)$$

(2) $$\mathrm{logit}\left(p_{i}\right)=\beta_{i,0}+\beta_{i,1}x_{i,1}+\beta_{i,2}x_{i,2}+\cdots +\beta_{i,k}x_{i,k}$$

where $\;y_{i}$ represents the occupying behavior indicator (=1 if the cyclists occupy motorized vehicle lanes and 0 otherwise) for the ith observation;  i=1,2,⋯n; n is the number of observations; $\;p_{i}$ represents the probability of occupying behavior; $\;X=\left[x_{i,1},x_{i,2},\cdot \cdot \cdot ,x_{i,k}\right]$ is the 1×k vector of explanatory variables; $\;\beta ={\left[\beta_{i,0},\beta_{i,1},\cdot \cdot \cdot ,\beta_{i,k}\right]}^{T}$ are the parameter vectors, and these parameters are allowed to vary across observations. In this model, the random parameters are assumed to be generally distributed as $\beta ∼N\left(\mu ,\Sigma \right)$ with $\mu ={\left[\mu_{1},\;\mu_{2}\cdots \mu_{k}\right]}^{T}\;and\;\Sigma =diag\left[\Sigma_{1},\Sigma_{2},\cdots \Sigma_{k}\right]$. The likelihood of the random parameter logit model is given by Equation (3) as follows:

(3) $$f\left(Y|\Theta \right)=\prod_{i=1}^{n}f\left(y_{i}|\beta \right)=\prod_{i=1}^{n}\left[P{\left(y_{i}=1\right)}^{y_{i}}{\left(1-P\left(y_{i}=1\right)\right)}^{1-y_{i}}\right]=\prod_{i=1}^{n}\left[{\left(\frac{exp\left(\beta_{i,0}+\beta_{i,1}x_{i,1}+\beta_{i,2}x_{i,2}+\cdot \cdot \cdot +\beta_{i,k}x_{i,k}\right)}{1+exp\left(\beta_{i,0}+\beta_{i,1}x_{i,1}+\beta_{i,2}x_{i,2}+\cdot \cdot \cdot +\beta_{i,k}x_{i,k}\right)}\right)}^{y_{i}}\times {\left(\frac{1}{1+exp\left(\beta_{i,0}+\beta_{i,1}x_{i,1}+\beta_{i,2}x_{i,2}+\cdot \cdot \cdot +\beta_{i,k}x_{i,k}\right)}\right)}^{1-y_{i}}\right]$$

where $Y=\left[y_{1},y_{2},\cdot \cdot \cdot ,y_{n}\right]\;$ includes observations; the vector of all parameters  Θ includes the random parameter vector β, random parameter mean vector μ, and random parameter variance vector Σ. Thus,  Θ is obtained as $\;\Theta =\left[\beta ,\;\mu ,\;\Sigma \right]$.

### 4.2. Full Bayesian Estimation

Before the model parameter estimation, the collinearity and correlation between explanatory variables were estimated using the Pearson correlation coefficient. If two variables were found to be significantly correlated in the correlation analysis, they were input into the model one by one while monitoring the overall fit of the model and the significance of the variables. Only variables with no significant correlations were retained in the model.

A Bayesian inference approach based on Markov chain Monte Carlo (MCMC) was adopted to simulate the posterior distribution Θ  [29,35]. It provides a more flexible modeling framework for solving the aforementioned complex model parameters. The Bayesian approach assumes that probability distributions can describe all the unknown parameters of the model as random variables. Based on the prior distribution of the parameters and sample observations, a posterior distribution of the parameters based on the sample observations can be inferred. Therefore, the posterior joint distribution of parameters  Θ can be estimated using the following function, Equation (4):

(4) $$f\left(\Theta |Y\right)=\frac{f\left(Y,\;\Theta \right)}{f\left(Y\right)}=\frac{f\left(Y|\Theta \right)\pi \left(\Theta \right)}{\int f\left(Y,\Theta \right)d\Theta }\propto f\left(Y|\Theta \right)\pi \left(\Theta \right)$$

where $f\left(\Theta |Y\right)$ denotes the posterior joint distribution of parameters  Θ conditional on dataset Y; and $f\left(Y,\;\Theta \right)$ represents the joint distribution of dataset Y and the model parameters  Θ. The term $f\left(Y|\Theta \right)$ denotes the likelihood conditional function on the parameters  Θ, specified by Equation (3). The function $\pi \left(\Theta \;\right)$ denotes the prior distribution of the parameters  Θ.

Owing to the lack of information on the random parameters, the non-informative prior distribution for  Θ is specified as follows Equations (5)–(7):

(5) $$\beta ∼N(\mu_{k},\Sigma_{k})$$

(6) $$\mu_{k}∼N\left(\bar{a_{k}},\bar{b_{k}}\right)$$

(7) $$\Sigma_{k}∼Inverse\;gamma\left(\bar{c_{k}},\bar{d_{k}}\right)$$

where all the priors of the mean of the random parameters follow the normal distribution, whereas the variance of the random parameters follows the inverse gamma distribution. The hyper-parameters in Equations (5)–(7) are set as expressed in Equation (8).

(8) $$\bar{a_{k}}=0,\;\bar{b_{k}}=10^{6},\;\bar{c_{k}}=0.001,\;\bar{d_{k}}=0.001$$

Based on the specification of the prior distributions for the parameters  Θ, the posterior joint distribution $\;f\left(\Theta |Y\right)$ can be derived as follows:

(9) $$f\left(\Theta |Y\right)\propto f\left(Y|\Theta \right)\pi \left(\Theta \right)=\prod_{i=1}^{n}f\left(\left(y_{i}|\beta \right)\right)\times \prod_{i=1}^{n}\overset{k}{\underset{k=0}{\prod }}N\left(\beta_{i,k}{|\mu }_{k},\Sigma_{k}\right)\times \prod_{k=0}^{k}N\left(\mu_{k}|\bar{a_{k}},\bar{b_{k}}\right)\times \prod_{k=0}^{k}IG\left(\Sigma_{k}|\bar{c_{k}},\bar{d_{k}}\right)$$

## 5. Results and Discussion

### 5.1. Comparison of Estimation Results

Both the standard logit model and Bayesian random parameter logit model were estimated to evaluate the impact of factors on COMB. OpenBUGS software was used as a modeling platform to calibrate the two models separately. Two Markov chains with different initial values were constructed for Bayesian inference. Finally, the two chains interact stably together, and the models converge. The performances of the two models were compared using the deviance information criterion (DIC).

Table 3 and Table 4 summarize the results of the standard logit model and the Bayesian random parameter logit model. The DIC of the standard logit model is 33,230, and the DIC of the Bayesian random parameter logit model is 33,210. The difference in the DIC is 20. El-Basyouny and Sayed [35] highlighted that a difference in DIC greater than 10 may allow a higher DIC model to be excluded. The model comparison results demonstrate that the proposed random parameter logit model is positively favored by empirical data. The standard deviation of the random parameter logit model is smaller than that of the standard logit model, confirming that accounting for unobserved heterogeneity among observations can improve the model fit. Subsequently, the odds ratio (OR) was employed to analyze the impact of factors on COMB.

### 5.2. Results of Cyclists’ Occupying Motorized Vehicle Lane Behavior at All Bike Facility Configurations

#### 5.2.1. Interpretation of Individual Characteristic Variables

Gender was found to be correlated with COMB. According to the OR listed in Table 4, the probability of male COMB is approximately 1.86 times that of females. This is consistent with previous studies showing that males are more inclined to engage in risky riding [29,36].

**Figure 4:**
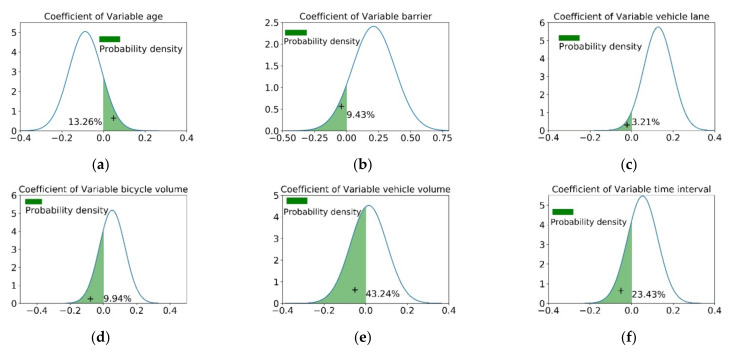
Distribution of parameters estimation for variables. (**a**) Distribution of parameters estimation for age; (**b**) Distribution of parameters estimation for barrier; (**c**) Distribution of parameters estimation for vehicle lane; (**d**) Distribution of parameters estimation for bicycle volume; (**e**) Distribution of parameters estimation for vehicle volume; (**f**) Distribution of parameters estimation for time interval.

Young cyclists had the highest probability of occupying motorized lane behavior and on the contrary old riders showed a negative correlation. For middle-aged cyclists, the parameter of the variable is subjected to a normal distribution whose mean value is −0.088 and whose standard deviation is 0.079. As shown in Figure 4a, 86.74% of middle-aged cyclists have a lower probability of occupying the motorized lane than young cyclists. In comparison, the other 13.26% preferred risky riding. This displays unobserved heterogeneous effects across individuals.

Bike type is significantly associated with COMB. As summarized in Table 4, the occupied motorized lane behavior of e-bike riders is 3.281 times higher than conventional bicycles, whereas that of e-scooter riders is 6.184 times higher than that of traditional bicycles. Electric two-wheeler riders have a higher probability of occupying the motorized vehicle lane. This may be because electric two-wheelers with higher velocity have solid requirements for more expansive horizontal traffic space, which mismatches poor riding conditions. According to the statistics in Table 2, the proportion of electric two-wheelers accounted for 86.7%. The significant change in the ratio of bike types resulted in a more aggressive riding behavior.

In addition, 61.61% of the light electric tricycles were cycled in the motorized vehicle lane, whereas 38.39% opted to cycle in the bike lane in the motorized vehicle lane. Because of the wide body of the tricycle, other cyclists have no space to drive and are forced to occupy the motorized vehicle lane. This should be taken seriously and given appropriate legal guidance.

#### 5.2.2. Road Geometric Design Variables

The dividing strip types between the motor and non-motorized vehicle lanes were significantly correlated with COMB. According to the OR listed in Table 4, for bike lanes with a greenbelt dividing strip as a reference variable, the result indicates that the occupancy probability of cyclists in bike lanes separated by marking is 6.931 times higher than that of non-motorized lanes separated by a greenbelt. Similarly, the occupancy probability of cyclists in the pedestrian–bicycle shared lane and mixed traffic are 5.618 times and 9.497 times higher than bike lanes isolated by a greenbelt, respectively. Additionally, the parameter of the barriers variable was normally distributed (0.212, 0.165). According to the parameter distribution in Figure 4b, it was found that the barriers dividing strip increase the probability of occupancy for 90.57% of riders, and for the remaining 9.43%, the probability of occupancy decreases. This is because that the bike lanes isolated by barriers often have insufficient space, and some riders occupy the motorized vehicle lane to avoid congestion and interference. This analysis shows that mixed traffic and bike lanes isolated by marking have the highest occupancy probability by cyclists, followed by pedestrian–bicycle lanes. The lowest occupancy probability is for non-motorized lanes isolated by physical facilities.

The bike lane width affects COMB. When 4.5 m wide lanes are selected as the reference variable, the occupancy probability of cyclists at 2.5 m and 3.5 m bike lanes are 3.053 times and 0.66 times greater, respectively. Usually, the wider the bike lane, the lower the occupancy rate of cyclists. However, the 3.5 m bike lane is the median opening road, and cyclists occupy the motorized lane to find opportunities to cross to the opposite side to reduce the detour directly to the destination. It is observed that when the road design provides the riders with the possibility of crossing, it is easy for them to occupy the motorized vehicle lane.

The explanatory variable of the vehicle lane number is associated with COMB. The vehicle lane number is set as one as the reference variable. The parameters of the variable with vehicle lane number 2 obeyed a normal distribution of (0.128, 0.069), indicating that the occupancy probability increases with an increase in vehicle lane number for 96.79% of the riders. It decreases with an increase in vehicle lane number for 3.21% of the riders, as shown in Figure 4c. When the number of motorized vehicle lanes is three, the occupancy probability is 2.901 times higher than when the vehicle lane number is one. In contrast, when the vehicle lane number is four, the parameter is −1.852, which exhibits a negative correlation. This is because 4-lane roads are usually urban arterials with high vehicle speeds, making them dangerous to ride on. It can be found that when the road grade is low, the loose conditions of motorized vehicle lanes provide the occupied space for cyclists. With the promotion of road grading, the danger of high-speed vehicles inhibits the possibility of such encroachment.

#### 5.2.3. Traffic Condition Variables

Bike volume is associated with COMB. According to the OR analysis in Table 4, the parameter of the medium bike volume is normally distributed with (0.053, 0.077), indicating that 90.06% of riders have a higher probability of being involved in COMB than low bike volume. In contrast, the remaining 9.94% have a lower probability of occupying motorized vehicle lane behaviors. This can be seen in Figure 4d. The result implies heterogeneous effects across the medium bike volume. Moreover, the occupancy probability of a high bike volume is 1.38 times that of a low bike volume.

The vehicle volume is related to COMB. As shown in Figure 4e, based on the OR analysis, the parameter of the medium vehicle volume is normally distributed with (0.015, 0.088). 43.24% of riders have a lower probability of COMB under medium vehicle volume than low vehicle volume. This indicates that the influence of medium vehicle volume on cycling behavior is weak. However, the occupancy probability of a high vehicle volume is 1.38 times that of a low vehicle volume.

On-street parking in a bike lane is negatively associated with COMB. Riders are only 0.36 times involved in occupying motorized vehicle lane behaviors at on-street parking in non-motorized lanes than without on-street parking. This was unexpected. After analyzing the data, we found that bike lanes with on-street parking have efficient widths, and on-street parking blocks the inference from pedestrians and other obstacles. This is conducive to the smoothness of riding. It also indicates that the disturbance of cycling is severe in China.

Temporary on-street parking is significantly and positively associated with COMB. Riders are 3.149 times more involved in occupying motorized vehicle lane behaviors at on-street parking in the bike lane than those without on-street parking. This is because the effective width of the current bike lane is mostly from 2.5 m to 4.5 m. Temporary on-street parking occupies the majority of the bike lane width. The space for non-motorized vehicles is sharply compressed or unavailable, and cyclists must occupy motorized vehicle lanes.

#### 5.2.4. Environmental Condition and Other Variables

Rainfall is negatively associated with COMB. This is because there are fewer rides on rainy days. Moreover, Daily recording time intervals are related to COMB. According to the OR analysis, the occupancy probability during the afternoon peak is 1.35 times higher than that during the morning peak. Moreover, as shown in Figure 4f, the parameter of the evening peak is normally distributed with (0.053, 0.073), indicating that the evening peak could increase the occupancy probability for 76.57% of riders, whereas for the other 23.43%, the probability of occupancy decreases. This may be related to the dark light, and some people are in a hurry to get home; others are cautious. This result implies heterogeneous effects across daily recording time intervals.

Manned riding is negatively associated with COMB. According to the OR analysis in Table 4, the occupancy probability of manned riding is 0.821 times that of independent riding. This may be because manned riding cyclists are cautious.

A working day is significantly correlated with COMB. According to the OR analysis, the occupancy probability on working days is 3.136 times higher than that of holidays. This intuitively has to do with less travel and more time during holidays.

These results show that bike types, dividing strip types, bike lane width, temporary parking, and workday are significantly related to COMB. This indicates that the intense encroachment of electric two-wheelers, available space conditions for occupancy, insufficient width of non-motorized lanes, external disturbances such as temporary parking, and the strong travel demand on weekdays are the leading causes of COMB.

### 5.3. Comparision Results of COMB According to Bicycle Facility Configurations

From the presented results, we found that the dividing strip between non-motorized lanes and motor vehicle lanes had the most significant impact on COMB. The occupancy probabilities of different types of dividing strips varied widely. However, there is still a lack of description of this discrepancy. Therefore, it is necessary to analyze the discrepancy in the factors influencing the behavior of cyclists occupying motorized vehicle lanes in each form of the dividing strip bike facility. The results of the estimation models for different bicycle facility configurations are presented to assess cyclist behavior in this section.

#### 5.3.1. Estimation of COMB at Greenbelt Dividing Strip

The results of the estimation are summarized in Table 5. The variables of male, e-bike, e-scooter and high bike volume are positively related to COMB. High vehicle volume, on-street parking, rainy weather, old riders, and manned riding are negatively associated with COMB. Among them, male sex, bike type, and rainy weather were the most significant factors.

As summarized in Table 5, in the bike lane with a greenbelt dividing strip, compared to young cyclists, 80.83% of middle-aged cyclists are less likely to occupy motorized lanes, whereas the remaining 19.17% have a higher probability of occupying motorized lanes. The low to medium bike volume increases the occupancy probability of 69.30% of cyclists and decreases the occupancy probability for the remaining 30.70%. Similarly, a low to medium vehicle volume decreases the occupancy probability of 95.27% of cyclists and increases the occupancy probability for the remaining 4.73%. Middle-aged, medium bike volume, and medium vehicle volume variables have unobserved heterogeneity. The results further confirm that the Bayesian random parameter logit model captures the unobserved heterogeneity effect, where individuals react differently to traffic conditions in the bike lane with a greenbelt dividing strip. It can be found that greenbelts constrain the impact of traffic volume variations.

#### 5.3.2. Estimation of COMB at Barriers Dividing Strip

As summarized in Table 6, the variables of male, e-bike, e-scooter, and high bike volume are positively related to COMB. This is consistent with the estimation of COMB at the greenbelt dividing strip. The old riders, manned, and evening peaks are negatively associated with COMB. Males and bike types are the most significant factor.

Middle-aged, medium bike volume, and vehicle lane number variables have unobserved heterogeneity. In the bike lane with barrier dividing strip, 73.42% of middle-aged cyclists were less likely to occupy motorized lanes compared with young cyclists; the remaining 26.57% are on the contrary. The low to medium bike volume increased the occupancy probability of 99.81% of cyclists and decreased the occupancy probability for the remaining 0.19%. Compared to vehicle lane number 2, the increase in the number of motorized lanes to three lanes improved the occupancy probability for 54.13% of cyclists and reduced the occupancy probability for the remaining 45.87%. The results further confirm that the Bayesian random parameter logit model captures the unobserved heterogeneity effect. Individuals have a different choice between the increase in bike volume and the available traffic space in the bike lane with barriers dividing strip.

#### 5.3.3. Estimation of COMB at Bike Lane with Marking Dividing Strip

As observed in Table 7, the variables of male, e-bike, e-scooter, and temporary parking are positively related to COMB. Old riders, on-street parking, and high motor volume are negatively associated with COMB. Bike type and temporary parking are the most significant factors.

Middle-aged, medium bike volume, afternoon peak, and manned riding variables have unobserved heterogeneity. In the non-motorized lane with marking, 85.38% of middle-aged cyclists are less likely to occupy motorized lanes compared with young cyclists, and the remaining 26.57% are more likely to occupy motorized lanes. The low to medium bike volume increased the occupancy probability of 81.72% of cyclists and decreased the occupancy probability for the remaining 18.28%. In addition, riding at the afternoon peak had a higher probability of occupying motorized lanes for 97.85% of riders, whereas the other 2.15% had a lower probability. This is intuitively due to the low vehicle volume in the afternoon, and cyclists are pursuing convenience and high speed; thus, they roam freely in the motorized vehicle lane. This also reflects the soft restrictive nature of the marking dividing strip for cyclists. Moreover, manned riding increased the occupancy probability of 57.88% of cyclists and decreased the occupancy probability for the remaining 42.12%. The analysis indicate that the marking dividing strip has low restrictions for cyclists. More relaxed conditions lead to a robust random selection behavior of cyclists. Cyclists react differently to medium bike volume, afternoon peak, and manned riding in the bike lane with a marking dividing strip.

#### 5.3.4. Estimation of COMB at Pedestrian–Bicycle Shared Lane

As summarized in Table 8, the variables of e-bikes, e-scooters, high bike volume, and afternoon peak are positively related to COMB. Old riders and high motor volume are negatively associated variables. Bike type and afternoon peak are the most significant factors.

Gender, old riders, medium bike volume, high vehicle volume, and manned riding variables were found to have heterogeneous effects, appearing in the estimates of random parameters in the statistical model. As summarized in Table 8, the occupancy probability increases by 61.26% for male cyclists compared to female cyclists on pedestrian–bicycle shared boards, and the remaining 38.74% decreases. Of the elderly cyclists, 99.83% are less likely to occupy motorized lanes in comparison with young cyclists, and the remaining 0.17% are more likely to be old cyclists. Moreover, a low to medium bike volume increases the occupancy probability of 81.72% of cyclists and decreases the occupancy probability for the remaining 18.28%. Similarly, the occupancy probability decreases with an increase in vehicle volume for 55.07% of the riders; it increases with vehicle volume for 44.93% of the riders. Manned riding increased the occupancy probability of 58.09% of cyclists and decreased the occupancy probability for the remaining 44.24%. The results revealed that individuals have different choices according to varying levels of the game combination of bike volume and vehicle volume in a pedestrian–bicycle shared lane. This is intuitive because the pedestrian–bicycle shared lane has significant pedestrian interference and poor continuity. The behavior of cyclists was randomly selected according to the individual and traffic conditions. This indicated cyclists had a relatively low acceptance of this bicycle facility.

#### 5.3.5. Estimation of COMB at Mixed Traffic

As summarized in Table 9, the variables of male, e-bike, e-scooter, and temporary parking are positively related to COMB. Bike type and temporary parking are the most significant factors. It is consistent with the estimation model in the bike lane with a marking dividing strip. There is a slight difference in that only the manned riding variable is a positive factor. This is because less vehicle volume in mixed traffic brings manned riding more confidence.

As listed in Table 9, the gender, middle-aged, medium bike volume, rainy, and manned riding variables have random parameters. In the mixed traffic non-motorized lane, 59.87% of males are more prone to risky riding than females. Moreover, 30.25% of middle-aged cyclists are less likely to occupy motorized lanes than young cyclists, whereas the remaining 69.75% are on the contrary. The occupancy probability of 50.12% for cyclists increases with an increase in the bike volume, whereas the remaining 49.88% decreases. Manned riding and rainy conditions increase the occupancy probability of 57.88% and 72.94% cyclists, and decrease the occupancy probability for the remaining 42.12% and 27.06%, respectively. In mixed traffic, each type variable exhibits unobserved heterogeneity, which shows the randomness of riding behavior under fewer constraint conditions.

## 6. Conclusions

This study investigated COMB at five types of bike lane facilities across different form dividing strips. In total, 34,631 riding samples were analyzed. Full Bayesian random parameter logit models were developed to explore the factors that significantly contribute to COMB. The unobserved heterogeneous effects associated with these observations were successfully captured.

The estimated model of all bike facility configurations exhibited five factors that significantly contributed to COMB, including bike types, dividing strip types, bike lane width, temporary parking, and workday. Moreover, bike lane width, bike volume, vehicle volume, barriers dividing strip, vehicle lane numbers, and time intervals have heterogeneous effects on the random parameter model.

Comparing the estimated model of five bike lane facilities across different dividing strips, we found that cyclists have the highest occupying probability for the bike lane with marking dividing strips and mixed traffic. The pedestrian–bicycle shared lane is second, whereas the bike lane with a physical dividing strip is the lowest.

Similarly, heterogeneity factors have been considered in detail for different dividing strip conditions and each one has its focus. It was found that physical dividing strips inhibit the effect of traffic volume variations. Therefore, the variables of the traffic conditions are heterogeneous. Moreover, individual according to traffic combinations have heterogeneous effects on the pedestrian–bicycle shared lane. Owing to pedestrian interference and poor continuity in the pedestrian–bicycle configuration, it brings the randomness of the selection of the cyclist. Finally, individuals have a heterogeneous reaction to variables such as bike volume, manned riding, time, and weather under the unrestrained bike lane with marking dividing strips and mixed traffic. We can conclude that the more open the bike lane conditions, the more possibilities for cycling behavior choices, and the more heterogeneous factors. Conversely, a physical dividing strip can restrain the heterogeneity of riding behavior. This can provide guidance on renovation of facility type.

The findings of this study can offer valuable insights into the underlying relationship between risky factors and COMB in five types of bike lanes. This contributes to the implementation of more effective countermeasures to reduce risky behavior. First, the speed of electric bicycles should be limited to no more than 25 km/h, which is actively promoted in China. Second, penalties for temporary illegal parking should be increased and the right of way for non-motor vehicles should be protected. Moreover, owing to the wide body and fast speed of electric bicycles, non-motorized lanes should be appropriately widened. Additionally, physically separated bicycle facilities reduce the interference between vehicles and bicycles and provide a safe cycling environment for cyclists. Improvements to physical isolation are recommended in the form of bicycle facilities. This facility type is recommended under what conditions, as well as the proper bike lane width, will be the focus of our next research.

Our study has certain limitations. The overall sample is sufficient, but limited by the road conditions of the city; data should be collected from more locations and cities to support the research. In addition, although our focus is on section data in this research, we realize that the continuous cycling environment also has an impact on cyclists’ road-occupying behavior, such as the frequency of obstacles. We will endeavor to continue this research to enhance the safety improvement of the cycling environment.

## Figures and Tables

**Table 1:** Road geometry at field data sites.

Road	Distance to Intersection (m)	Dividing Strip	Bike Lane Width (m)	Vehicle Lane Number	On-Street Parking
Jianshe Road	200	Greenbelt	4.5	4	No
Guangming Road	100	Greenbelt	3.5	2	Yes
Kaiyuan Road (N)	100	Marking	2.5	2	Yes
Kaiyuan Road (S)	150	Barriers	2.5	2	No
Kuanggong Road	150	Marking	2.5	3	No
Lingyun Road	100	Barriers	2.5	3	No
Zhanbei Road	100	Pedestrian–bicycle shared	2.5	1	No
Shuguang road	100	Mixed	1.5	1	Yes

**Table 2:** Descriptive statics of explanatory variables.

	Variable	Definition	Frequency	Percentage
Individual characteristics	Gender	0: Female 1: Male	18,014 16,617	52.0% 48.0%
	Age	1: Young: <35 2: Middle-aged: 35–60 3: Old: >60	6362 24,363 3906	18.4% 70.4% 11.3%
	Bike type	1: C-bike 2: E-bike 3: E-scooter 4: Electric tricycle	1944 10,656 19,348 2683	5.6% 30.8% 55.9% 7.7%
Road Geometric Design	Bike lane width(m)	1: 4.5 2: 2.5 3: 3.5 4: 1.5	5052 22,518 5650 1411	14.6% 65.0% 16.3% 4.1%
	Vehicle lane number	1: 2 2: 3 3: 4 4: 1	16,925 7788 5052 4866	48.9% 22.5% 14.6% 14.1%
	Dividing strip types	1: Green belt 2: Barriers 3: Marking 4: Pedestrian–bicycle shared 5: Mixed	10,702 9433 9630 3455 1411	30.9% 27.2% 27.8% 10.0% 4.1%
Traffic condition	Bike volumeveh/(5 min·m)	1: Low: ≤33 2: Medium:34–53 3: High: ≥53	7826 10,689 16,116	22.6% 30.9% 46.5%
	Vehicle volumeveh/(5 min·lane)	1: Low: ≤19 2: Medium: 20–38 3: High: ≥39	5239 26,818 2574	15.1% 77.4% 7.4%
	On-street parking	0: No 1: Yes	23,158 11,473	66.9% 33.1%
	Temporary parking	0: No 1: Yes	31,878 2 753	92.1% 7.9%
Environment condition	Weather	1: Sunny 2: Cloudy 3: Rainy	17,409 16,195 1027	50.3% 46.7% 3.0%
	Time intervals	1: Morning 2: Noon 3: Evening	12,352 10,561 11,718	35.7% 30.5% 33.8%
Others	Manned riding	0: No	28,438	82.1%
		1: Yes	6193	17.9%
	Workday	0: No	10,701	29.2%
		1: Yes	23,930	70.8%

**Table 3:** Estimation from the standard logit model.

Variable		Mean	S.D.	2.5%	97.5%
Intercept		−4.675	0.748	−6.250	−3.594
Gender	Male vs. Female	0.621	0.031	0.560	0.681
Age	Middle-aged vs. Young	−0.088	0.038	−0.163	−0.013
	Old vs. Young	−0.334	0.059	−0.451	−0.218
Bike type	E-bike vs. C-bike	1.189	0.082	1.031	1.352
	E-scooter vs. C-bike	1.822	0.080	1.670	1.982
	Tricycle vs. C-bike	2.161	0.091	1.985	2.341
Dividing strip	Barriers vs. green belt	0.211	1.538	0.314	0.109
	Marking vs. green belt	2.250	3.604	−2.988	8.519
	Pedestrian–bicycle vs. green belt	1.724	6.997	−11.41	10.52
	mixed vs. green belt	1.937	1.536	−0.749	4.462
bike lane width	2.5 vs. 4.5	1.115	0.740	−0.008	2.673
	3.5 vs. 4.5	−0.416	1.616	−3.677	2.660
	1.5 vs. 4.5	4.142	8.362	−7.172	18.09
vehicle lane number	2 vs. 1	0.127	0.080	−0.029	0.286
	3 vs. 1	1.065	1.582	−2.578	3.948
	4 vs. 1	−1.851	4.769	−9.742	5.271
bike volume	Middle vs. Low	0.052	0.054	−0.053	0.157
	High vs. low	0.322	0.066	0.196	0.452
Vehicle volume	Middle vs. Low	0.014	0.004	−0.021	0.053
	High vs. low	−0.213	0.097	−0.405	−0.023
On-street parking	Yes vs. No	−1.022	0.098	−1.209	−0.826
Temporary parking	Yes vs. No	1.147	0.055	1.039	1.253
Weather	Cloudy vs. sunny	−0.231	0.073	−0.374	−0.089
	Rainy vs. sunny	−0.712	0.152	−1.010	−0.418
Time interval	Noon vs. morning	0.299	0.038	0.224	0.372
	Evening vs. morning	0.053	0.037	−0.020	0.124
Others	Manned riding	−0.197	0.040	−0.276	−0.197
	Work day	1.141	1.321	−1.668	3.475
DIC		33,230			

**Table 4:** Estimation from the Bayesian random parameters logit model.

Variable		Mean	S.D.	2.5%	97.5%	OR
Intercept		−4.680	0.034	0.017	0.084	
Gender	Male vs. female	0.621	0.026	0.569	0.674	1.860
Age	Middle-aged vs. young	−0.088	0.029	−0.145	−0.030	0.916
	S.D. of parameter distribution	0.079	0.113	0.020	0.292	
	Old vs. young	−0.333	0.040	−0.414	−0.252	0.716
Bike type	E-bike vs. C-bike	1.188	0.035	1.117	1.261	3.281
	E-scooter vs. C-bike	1.822	0.034	1.751	1.892	6.184
	Tricycle vs. C-bike	2.161	0.040	2.078	2.243	8.680
Dividing strip	Barriers vs. green belt	0.212	0.050	0.315	0.110	1.236
	S.D. of parameter distribution	0.165	0.052	0.020	0.196	
	Marking vs. green belt	2.251	0.055	2.140	2.362	9.497
	Pedestrian–bicycle vs. green belt	1.726	0.063	1.602	1.858	5.618
	Mixed vs. green belt	1.936	0.048	1.834	2.032	6.931
bike lane width	2.5 vs. 4.5	1.116	0.069	0.987	1.252	3.053
	3.5 vs. 4.5	−0.415	0.070	−0.553	−0.274	0.660
	1.5 vs. 4.5	4.140	0.068	3.999	4.279	62.803
vehicle lane number	2 vs. 1	0.128	0.035	0.058	0.198	1.136
	S.D. of parameter distribution	0.069	0.061	0.020	0.217	
	3 vs. 1	1.065	0.057	0.948	1.181	2.901
	4 vs. 1	−1.852	0.051	−1.954	−1.749	0.157
bike volume	Middle vs. Low	0.053	0.033	−0.013	0.120	1.055
	S.D. of parameter distribution	0.077	0.107	0.020	0.287	
	High vs. low	0.324	0.035	0.253	0.394	1.383
Vehicle volume	Middle vs. low	0.015	0.004	−0.027	0.058	1.015
	S.D. of parameter distribution	0.088	0.138	0.021	0.333	
	High vs. low	−0.212	0.050	−0.316	−0.106	0.809
On-street parking	Yes vs. no	−1.021	0.041	−1.103	−0.938	0.360
Temporary parking	Yes vs. no	1.147	0.044	1.058	1.238	3.149
Weather	Cloudy vs. sunny	−0.230	0.039	−0.309	−0.149	0.795
	Rainy vs. sunny	−0.711	0.072	−0.862	−0.556	0.491
Time interval	Noon vs. morning	0.299	0.028	0.244	0.356	1.348
	Evening vs. morning	0.053	0.028	−0.003	0.110	1.055
	S.D. of parameter distribution	0.073	0.111	0.019	0.275	
Others	Manned riding vs. no	−0.197	0.034	−0.265	−0.129	0.821
	Work day vs. no	1.143	0.073	1.004	1.288	3.136
DIC		33,210				

**Table 5:** Estimation from the Bayesian random parameters logit model at bike lanes separated with greenbelt.

Variable		Random Parameter Logit Model				
		Mean	S.D.	2.5%	97.5%	OR
	Intercept	−3.148	0.101	−3.354	−2.933	
Individual characteristic	Gender					
	Male vs. famle	0.906	0.056	0.792	1.021	2.475
	Age					
	Middle-aged vs. young	−0.095	0.054	−0.204	−0.095	0.909
	S.D. of parameter distribution	0.109	0.166	0.022	0.436	
	Older vs. young	−0.271	0.070	−0.418	−0.122	0.762
Road Geometric Design	Bike type					
	E-bike vs. C-bike	0.450	0.064	0.316	0.581	1.568
	E-scooter vs. C-bike	1.067	0.059	0.944	1.188	2.907
	Tricycle vs. C-bike	1.200	0.073	1.041	1.352	3.320
Traffic condition	bike volume					
	Middle vs. low	0.057	0.055	−0.057	0.170	1.059
	S.D. of parameter distribution	0.113	0.461	0.022	0.428	
	High vs. low	0.414	0.068	0.269	0.554	1.513
	Vehicle volume					
	Middle vs. low	−0.239	0.085	−0.416	−0.066	0.787
	S.D. of parameter distribution	0.143	0.277	0.024	0.613	
	High vs. low	−0.340	0.100	−0.553	−0.126	0.712
	On-street parking	−0.477	0.061	−0.602	−0.350	0.620
Environment condition and others	Weather					
	Cloudy vs. sunny	−0.304	0.054	−0.416	−0.194	0.738
	Rainy vs. sunny	−1.813	0.114	−2.065	−1.565	0.163
	Time interval					
	Noon vs. morning	0.331	0.053	0.221	0.441	1.392
	Evening vs. morning	0.336	0.060	0.211	0.460	1.399
	Manned riding	−0.539	0.068	−0.681	−0.399	0.583

**Table 6:** Estimation from the Bayesian random parameters logit model at bike lanes separated with barriers.

Variable		Random Parameter Logit Model				
		Mean	S.D.	2.5%	97.5%	OR
	Intercept	−4.536	0.082	−4.698	−4.359	
Individual characteristic	Gender					
	Male vs. famle	1.029	0.049	0.931	1.129	2.798
	Age					
	Middle-aged vs. young	0.067	0.049	−0.035	0.166	1.069
	S.D. of parameter distribution	0.107	0.163	0.022	0.419	
	Older vs. young	−0.140	0.070	−0.290	0.005	0.870
	Bike type					
	E-bike vs. C-bike	1.043	0.057	0.920	1.158	2.838
	E-scooter vs. C-bike	1.717	0.053	1.603	1.821	5.568
	Tricycle vs. C-bike	2.147	0.063	2.014	2.274	8.559
Road Geometric Design	vehicle lane					
	3 vs. 2	0.065	0.065	−0.070	0.198	1.067
	S.D. of parameter distribution	0.627	0.119	0.024	2.129	
Traffic condition	Bike volume					
	Middle vs. yow	0.422	0.061	0.295	0.550	1.525
	S.D. of parameter distribution	0.145	0.234	0.022	0.429	
	High vs. low	0.345	0.061	0.219	0.472	1.412
Environment conditionand others	time interval					
	Noon vs. morning	0.438	0.046	0.345	0.531	1.549
	Evening vs. morning	−0.629	0.049	−0.730	−0.530	0.533
	Manned riding	−0.368	0.066	−0.503	−0.234	0.692

**Table 7:** Estimation from the Bayesian random parameters logit model at bike lanes separated with marking.

Variable		Random Parameter Logit Model				
		Mean	S.D.	2.5%	97.5%	OR
	Intercept	−0.887	0.073	−1.035	−0.736	
individual characteristic	Gender					
	Male vs. female	0.435	0.039	0.356	0.514	1.546
	Age					
	Middle-aged vs. young	−0.100	0.041	−0.183	−0.016	0.905
	S.D. of parameter distribution	0.095	0.166	0.021	0.378	
	Older vs. Young	−0.532	0.054	−0.644	−0.420	0.588
	Bike type					
	E-bike vs. C-bike	1.262	0.045	1.170	1.354	3.532
	E-scooter vs. C-bike	1.970	0.043	1.881	2.057	7.171
	Tricycle vs. C-bike	2.005	0.053	1.894	2.115	7.426
Traffic condition	bike volume					
	Middle vs. low	0.095	0.060	−0.031	0.224	1.100
	S.D. of parameter distribution	0.105	0.253	0.022	0.415	
	High vs. low	0.137	0.061	0.264	0.005	1.146
	Vehicle volume					
	Middle vs. low	−0.271	0.048	−0.368	−0.174	0.763
	On-street parking	−1.092	0.040	−1.174	−1.011	0.336
	Temporary parking	1.111	0.045	1.018	1.203	3.037
Environment condition and others	Time interval					
	Noon vs. morning	0.182	0.042	0.266	0.097	1.199
	S.D. of parameter distribution	0.090	0.197	0.021	0.335	
	Evening vs. morning	0.198	0.042	0.113	0.284	1.219
	Manned riding	−0.114	0.051	−0.217	−0.010	0.892
	S.D. of parameter distribution	0.574	0.142	0.024	2.132	

**Table 8:** Estimation from the Bayesian random parameters logit model at pedestrian–bicycle shared lane.

Variable		Random Parameter Logit Model				
		Mean	S.D.	2.5%	97.5%	OR
	Intercept	−3.885	0.086	−4.06	−3.706	
Individual characteristic	Gender					
	Male vs. female	0.205	0.064	0.071	0.335	1.228
	S.D. of parameter distribution	0.716	0.261	0.024	2.267	
	Age					
	Middle-aged vs. young	−0.321	0.061	−0.451	−0.195	0.726
	Older vs. Young	−0.340	0.075	−0.499	−0.179	0.711
	S.D. of parameter distribution	0.116	0.193	0.023	0.475	
	Bike type					
	E-bike vs. C-bike	1.654	0.072	1.504	1.8	5.228
	E-scooter vs. C-bike	2.241	0.068	2.100	2.378	9.403
	Tricycle vs. C-bike	3.658	0.078	3.492	3.913	38.784
Traffic condition	Bike volume					
	Middle vs. low	0.165	0.065	0.030	0.301	1.180
	S.D. of parameter distribution	0.120	0.215	0.023	0.470	
	High vs. low	0.186	0.076	0.027	0.186	1.204
	Vehicle volume					
	High vs. middle	−0.105	0.067	−0.244	0.035	0.900
	S.D. of parameter distribution	0.824	0.343	0.024	2.257	
Environment condition and others	time interval					
	noon vs. morning	1.168	0.062	1.039	1.297	3.216
	evening vs. morning	0.557	0.064	0.424	0.691	1.745
	Manned riding	0.077	0.085	−0.105	0.254	1.080
	S.D. of parameter distribution	0.531	0.426	0.026	2.596	

**Table 9:** Estimation from the Bayesian random parameters logit model in mixed traffic.

Variable		Random Parameters Logit Model				
		Mean	S.D.	2.5%	97.5%	OR
	Intercept	−1.458	0.010	−1.662	−1.243	
individual characteristic	Gender					
	Male vs. female	0.263	0.086	0.084	0.443	1.301
	S.D. of parameter distribution	1.051	0.384	0.025	2.557	
	Age					
	Middle-aged vs. young	−0.075	0.086	−0.259	0.101	0.928
	S.D. of parameter distribution	0.145	0.257	0.024	0.615	
	Older vs. young	−0.442	0.104	−0.666	−0.226	0.643
	Bike type					
	E-bike vs. C-bike	1.428	0.077	1.261	1.587	4.170
	E-scooter vs. C-bike	1.856	0.077	1.689	2.017	6.398
	Tricycle vs. C-bike	1.674	0.091	1.478	1.864	5.333
Traffic condition	bike volume					
	Middle vs. low	0.002	0.096	−0.210	0.205	1.002
	S.D. of parameter distribution	0.682	0.895	0.025	2.674	
	temporary parking	3.493	0.423	2.585	4.465	32.884
Environment condition and others	weather					
	rainy vs. sunny	0.564	0.138	0.269	0.859	1.758
	S.D. of parameter distribution	0.923	0.134	0.027	3.583	
	time interval					
	noon vs. morning	0.876	0.084	0.697	1.054	2.401
	evening vs. morning	0.865	0.087	0.680	1.048	2.374
	Manned riding	0.253	0.108	0.025	0.484	1.288
	S.D. of parameter distribution	0.920	0.231	0.026	3.123	

## Data Availability

The data used to support the findings of this study are available from the corresponding author upon request.
